# In-situ monitoring of an organic sample with electric field determination during cold plasma jet exposure

**DOI:** 10.1038/s41598-020-70452-w

**Published:** 2020-08-12

**Authors:** Elmar Slikboer, Ana Sobota, Enric Garcia-Caurel, Olivier Guaitella

**Affiliations:** 1 LPP, CNRS, Ecole Polytechnique, Sorbonne Universite, IP-Paris, 91128 Palaiseau, France; 2 Department of Applied Physics, EPG, Eindhoven University of Technology, Eindhoven, The Netherlands; 3 LPICM, CNRS, Ecole Polytechnique, IP-Paris, 91128 Palaiseau, France

**Keywords:** Optical sensors, Imaging and sensing, Imaging techniques, Applied physics, Plasma physics

## Abstract

Pockels-based Mueller polarimetry is presented as a novel diagnostic technique for studying time and space-resolved and in-situ the interaction between an organic sample (a layer of onion cells) and non-thermal atmospheric pressure plasma. The effect of plasma is complex, as it delivers electric field, radicals, (UV) radiation, non-uniform in time nor in space. This work shows for the first time that the plasma-surface interaction can be characterized through the induced electric field in an electro-optic crystal (birefringence caused by the Pockels effect) while at the same moment the surface evolution of the targeted sample is monitored (depolarization) which is attached to the crystal. As Mueller polarimetry allows for separate detection of depolarization and birefringence, it is possible to decouple the entangled effects of the plasma. In the sample three spatial regions are identified where the surface evolution of the sample differs. This directly relates to the spatial in-homogeneity of the plasma at the surface characterized through the detected electric field. The method can be applied in the future to investigate plasma-surface interactions for various targets ranging from bio-films, to catalytic surfaces and plastics/polymers.

## Introduction

Non thermal (cold) plasmas have been used at atmospheric pressure with great initial success for various applications e.g. biomedical treatment, surface functionalization of polymers and sterilization of various surfaces^1–3^. The plasma used for these applications is called cold since only the electrons reach elevated temperatures while the heavy particles stay at several hundred Kelvin. As a result there is a rich chemistry (e.g. reactive oxygen and nitrogen species in air) that can be achieved close to the treated surface at low input power (several Watts) used to generate the plasma. The plasma surface interaction however is difficult to study and new diagnostic techniques are necessary since established techniques used for low-pressure plasmas like (standard) ellipsometry are difficult to apply. This is because at atmospheric pressure the discharges are usually non-homogeneous in space and time in the form of ionization waves and additionally the complex samples (e.g. cells, tissues or fabrics) would cause a certain degree of depolarization of the probing polarization light beam which cannot be accessed using ellipsometry. Usually, the plasma and the surface are examined separately but this is far from ideal to characterize the plasma-surface interaction which is needed to further the research in these areas to eventually allow for a better control of the desired treatments.

This paper will introduce Mueller polarimetry as a novel optical diagnostic technique that allows monitoring of the surface evolution of the targeted material while characterizing the interaction by capturing images of the induced electric field due to deposited surface charges. This technique has been used in the past for the examination of various tissues^4–6^, however it has never been used for tissues in-situ during direct plasma exposure as we show in this work. A diagnostic with that capability would be very useful since different types of samples and various plasma devices/exposures can be investigated and compared through the induced electric field. This simultaneous examination is possible since Mueller polarimetry allows for partial depolarization of the probing light beam, making it distinctively different from other well established optical techniques, such as spectroscopic ellipsometry^7^.

The presented technique follows previous works where electro-optic materials under plasma exposure are examined (in transmission with a single wavelength) with Mueller polarimetry^8–11^. Mueller polarimetry is the optical investigation of materials by measuring their $4\times 4$ Mueller matrix, following the Stokes description of polarized light. The Mueller matrix describes the full optical response of the sample to polarized light in terms of reflection, transmission or scattering depending on the measurement conditions. Therefore, it incorporates information about the diattenuation properties of the material, together with the birefringence and depolarization.

Electro-optic targets are used since their refractive index changes with the externally induced electric field, according to the electro-optic effect (first order Pockels or second order Kerr effect). By analyzing the birefringence that the polarized light beam experiences while traveling through the target, the conditions to which the target are exposed to are revealed, in terms of the induced electric field by the plasma^8^. Other plasma diagnostic methods like Stark shift of forbidden Helium lines or second harmonic generation obtain the electric field from the plasma in the gas phase^12–15^. Although this information is important regarding the propagation of the ionization waves, it cannot be used for extrapolation to obtain what the target experiences since this is strongly dependent on the surface charges that are deposited on it^10,11,16^. Although a specific electro-optic target is used to study the induced electric field, numerical work^17^ indicates that the induced electric field should be generally comparable to targets at floating potential with dielectric constant higher than 4 and a similar thickness of 0.5 mm.

In this work, we show for the first time the possibility of studying the interaction of a plasma with a complex sample, i.e. a biological tissue, with the help of a electro-optic crystal. The complexity of the biological sample consists of its structure, made mainly of cells. Due of this heterogeneous structure the sample scatters light to some extent. Scattered light when combined with direct light in the detector becomes partially depolarized. Depolarization of light is usually seen as a drawback, but in the case discussed in this paper it turns out to be an advantage since it is related to scattered light and the latter is influenced by the structure of the sample. By capturing the depolarization, the surface evolution of the sample under plasma exposure is monitored while the birefringence still allows for the characterization of the discharge by imaging the induced electric field. The electric field is retrieved from the birefringent properties of the electric optic BSO (Bi$_{12}$SiO$_{20}$) target meaning it is the electric field within the crystal and not necessarily the same as experienced by the complex organic sample placed on top of the BSO. It allows to characterize in-situ the inhomogeneity of the plasma surface interaction through the surface charge deposition.

The complex sample which is used as a test case example is a single layer of epidermal cells of an onion (Allium cepa). This has been chosen because it is easily obtained, non-hazardous, the experiments are repeatable, and the cells of the onion are relatively large (100–300 $\upmu$m). The single layer of onion cells will be examined under exposure of guided ionization waves produced by an atmospheric pressure plasma jet. The non-thermal kHz-driven discharges generated by the jet are used regularly for applications ranging from biomedical treatment of cells and wounds to surface modifications of plastics and glasses.

**Figure 1:**
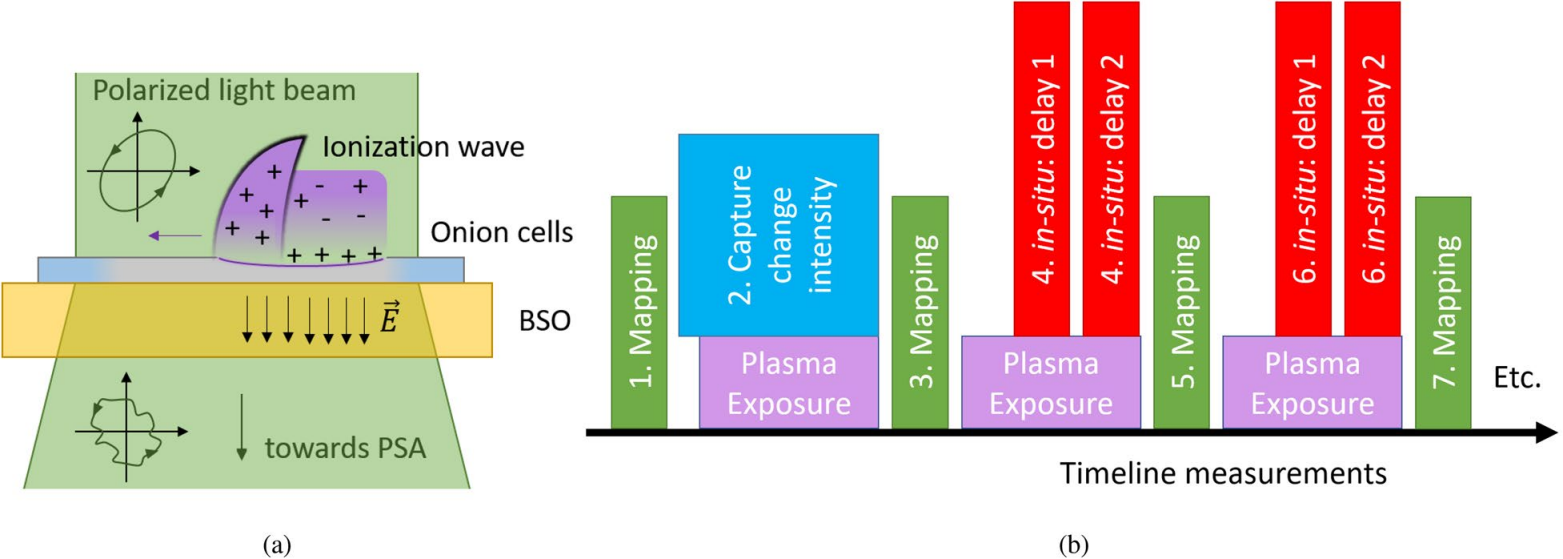
(**a**) Shows a schematic overview of the examination. (**b**) Shows the experimental timeline used during the examination of the combined sample, including the two different approaches. The first, shown in green, consists of a mapping done before and after plasma exposure. The second, shown in red, consists of the in-situ characterization of the interaction by measuring twice a time-resolved Mueller matrix under continuous plasma exposure to obtain the electric field.

A schematic is shown in Fig. 1a, showing the propagation on the onion cells of the ionization wave generated by the plasma jet after impact on the target. The interaction causes local changes (monitored through depolarization) to the onion cells due to etching, dewetting, and deposition of charges. The latter induces electric fields and the electric field in the BSO material is captured by looking at the changes in birefringence using the polarized light beam.

For these applications it is often difficult to speak of a strict quantification or characterization of the plasma-surface interaction. This is because the plasma has a synergistic effect consisting of radical species that impact the studied sample^18–20^, electric fields that are generated, (UV) radiation, heat that is delivered to the sample, and the impact of charged species^21–23^. This has lead to many applications in which a cold plasma is considered to be beneficial, but also has lead to difficulties to define some kind of “*dose*” that the plasma “*delivers*” to the surface. The quantification of the induced electric field is important because it relates to the surface charges that are deposited and it indicates the area where the direct interactions between the plasma and the surface takes place. Surface chemistry processes related to the electric field experienced by the target and also electro-poration or electrophoresis could play a part in the transport of aqueous species or DNA through membranes or cells^24^.

## Results

As discussed in the Methods section, the plasma surface interaction is examined following two approaches shown in Fig. 1b. Approach 1 follows the optical examination of the sample before and after plasma exposure while with approach 2 the examination is done in-situ during plasma exposure. During the first moments of plasma exposure of a “*fresh*” onion sample it was found that significant changes occur that render it impossible to examine the Mueller matrix of the sample in in-situ mode and thus the characterization of the plasma-surface interaction. To examine what happens during this initial moments simply the light intensity reaching the detector is monitored in time. These results are shown first.

### Initial surface changes by plasma impact

**Figure 2:**
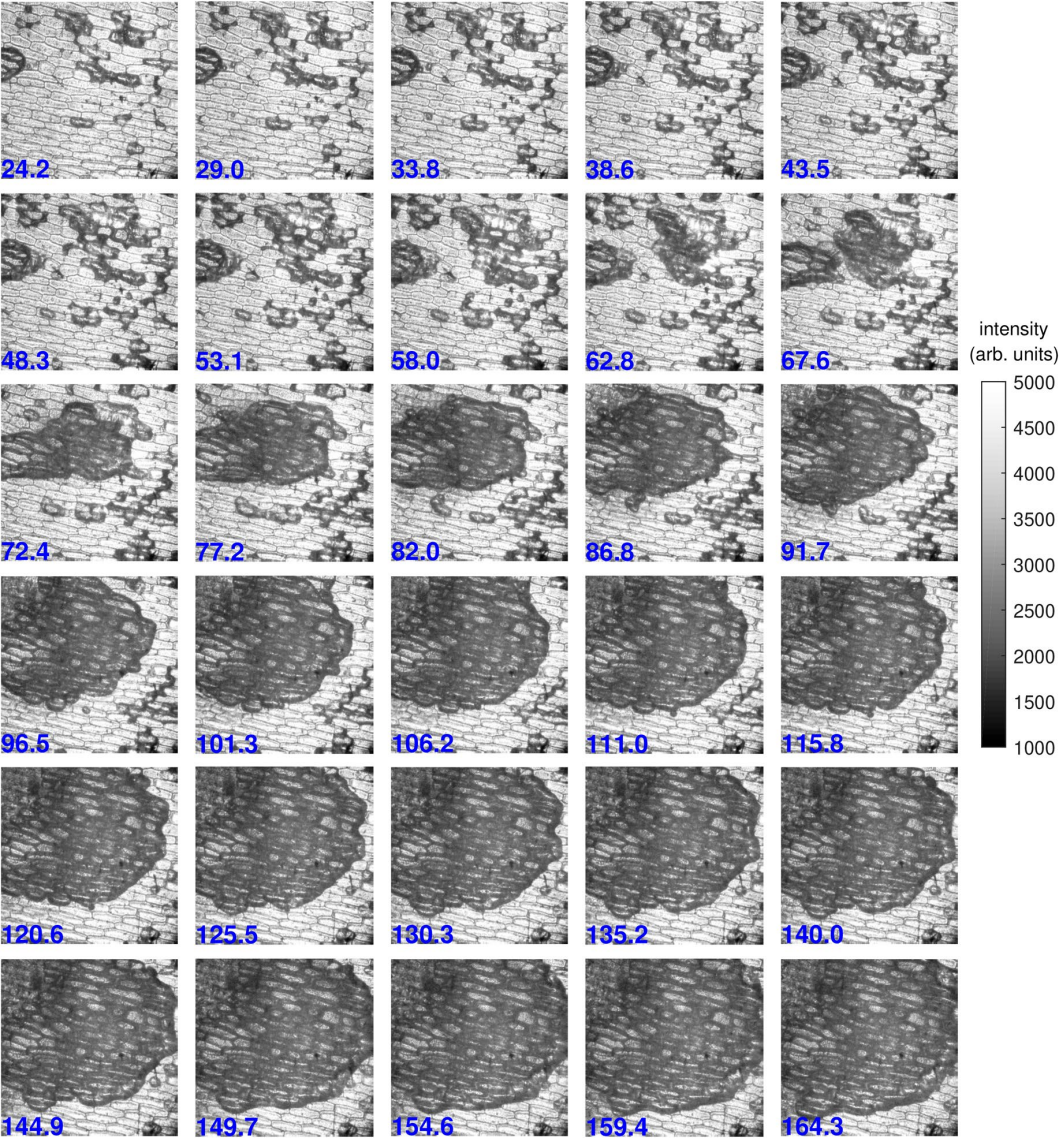
Change of intensity in time reaching the detector (while the PSA and PSG are in neutral orientation) showing the dewetting of a *fresh/new* onion sample^25^. At 15 s (time indication given in blue in seconds) the helium flow is started and at 45 s the AC voltage is applied. The plasma jet is oriented on the right-hand side and the images are $1.5\times 1.5$ mm$^2$ in size.

From the third layer of an onion, a single layer of cells on the outer facing side are carefully removed and placed upon the electro-optic BSO crystal. The intensity reaching the iCCD detector is captured continuously while the liquid crystals of the PSG and PSA are in neutral orientation. This means that quantitative polarimetric information cannot be obtained from the captured intensity, but changes that occur still show an interesting evolution of the sample under plasma exposure. Every acquisition consists of 50 frames taken with a 10 $\upmu$s exposure time, which takes 0.8 second to capture and another 0.8 second to save. Figure 2 shows every third image that is obtained, i.e. every 4.8 s. These results are part of the PhD thesis of Slikboer^25^. The time (in s) at which the image is obtained is shown in blue. The plasma exposure is continued until 8 min.

The spatial decrease of intensity suggests that a dewetting takes place caused by the plasma interaction. In a “*fresh*” sample, a liquid thin layer can act as refractive index matching medium between the complex sample and the air. This provides a clear image of the onion cell structure. When the liquid layer is removed, the transmission decreases because the refractive index between the sample and the air becomes higher, the latter causes the light to be scattered more efficiently, which reduces the flux of photons reaching the detector.

The light emission from the plasma impact area indicates a direct interaction with characteristic length scales of approximately $1.5\times 0.5$ mm$^2$, as will be confirmed by the electric field measurements done with the second approach. The heating of the surface by this plasma jet remains low, with a maximum temperature elevation of approximately 15 degrees^9^. As a result, the dewetting cannot be solely explained due to evaporation caused by the heating.

The interaction between an atmospheric pressure plasma jet and a liquid thin layer has been studied in the past and it was found that several effects can play a role. Firstly, the plasma exposure can cause changes in the surface tension of the liquid layer which can induce Marangoni flows^26^. The surface can also be deformed when the plasma is not ignited and only a gas jet is impinges the liquid layer^27^. Furthermore, surface charges can play a role because the electric field which is generated by them could enable dielectricphoresis^28^. Since the changes of intensity shown in Fig. 2 occur only with the impact of the ionization waves present and not with a helium gas flow alone, this could be an important aspect of the interaction. Lastly, the influence of infrared laser spots on liquid thin films has been studied in the past^29,30^, where it was shown that the occurrence, timing and velocity of dewetting and rupture of the film is influenced by the energy and spot size.

### Approach 1: Before and after exposure to plasma

Following the first approach, the plasma-surface interaction is investigated by using Mueller polarimetry for the optical investigation of the sample before and after exposure to the plasma. The Mueller matrix is measured successively on nine different locations of the sample, located in a 3$\times$3 square grid with 1.5 mm width. The central location corresponds with the field of view of the frames shown in Fig. 2. The sample can be moved and returned to the original location with a high accuracy by using motorized translation stages. The plasma impact occurs primarily in the central location of the image, with the plasma jet located on the right-hand side at a 7 mm distance. Any propagation of the plasma at the surface will be towards the left, as will be shown with the results from approach 2.

**Figure 3:**
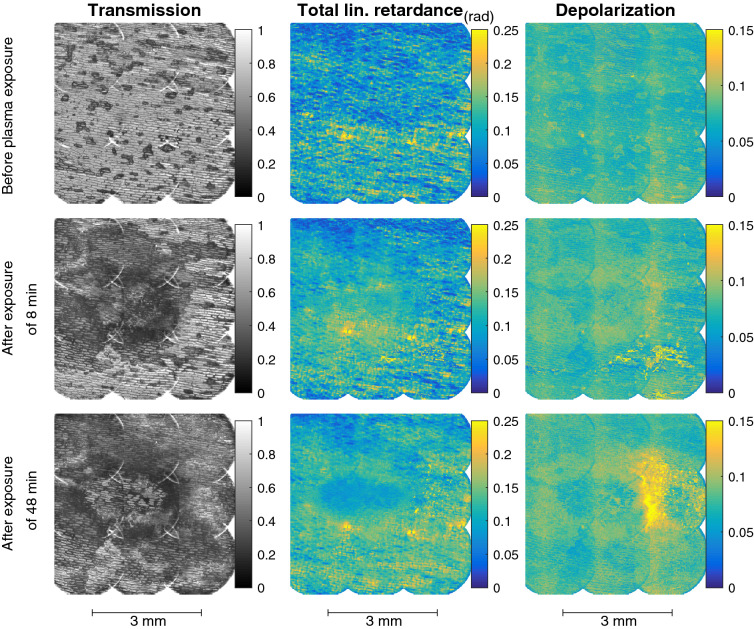
Obtained maps of the transmission, total retardances (rad) and total depolarization of the combined sample of BSO and the single layer of onion cells^25^. The optical properties are obtained before any plasma interaction and after 8 and 48 min of total exposure. The mapping is done by measuring the Mueller matrices while moving the sample along a $3\times 3$ grid.

The optical properties with the 9 different (sub)images are combined and at the edges where the data overlaps the average is taken. The transmission, total linear retardance and total depolarization of the sample are specifically looked at, and the results can be seen in Fig. 3 respectively before and after 8 and 48 min of plasma exposure.

At first sight, the most noticeable changes due to the plasma exposure are visible for the overall transmission of the sample. Before plasma exposure the structure of the single onion cell layer is clearly visible. After 8 min of exposure the effect of dewetting is significant. This relates to the changes observed in Fig. 2. The most visible changes are seen in the center and towards the top left, due the impact direction of the plasma jet. The bottom and right part of the sample are affected after a later time, as shown by the transmission obtained after 48 min of exposure. At this time-scale there is also a clear suggestion that etching has taken place in the central impact region.

There is also a visible effect due to the plasma exposure for the total linear retardance and the depolarization. Before plasma exposure, the observed linear retardance could be caused by the liquid thin layer that is present in addition to the membrane structure, orientation, and chemical composition of the onion cells. When the liquid thin layer is removed due to the plasma impact, a slight increase of the linear retardance is observed.

Also, the occurrence of etching at longer time-scales is visible since the linear retardance decreases in the region where the plasma interacts directly to the surface of the complex sample. The decrease is directly related to the thickness of the sample since the optical examination is done in transmission, meaning the total phase retardance scales linearly with it. The area where the retardance has decreased after 48 min is regarded as the direct impact region of the plasma exposure. This is confirmed with approach 2 by the imaging of the induced electric fields.

Regarding depolarization, the observed changes are most significant in the area surrounding the impact region. This mapping region relates to the area within the transmission maps where the strongest decrease has been observed. When the transmission of a sample decreases, it means that automatically more light has been scattered away. This means it is related to the (increasingly severe) dewetting of the surface. In addition there is a region to the right of the impact point where the depolarization has reached the highest observed 0.15 value. This sharp increase is spatially located on the side of the impact point which is close the plasma jet.

Upon repeating the experiments with new onion cells exposed to the plasma jet in identical conditions, all the discussed observed changes are reproducible (dewetting, darkening of the sample, change of linear retardance and depolarization) except for this sharp increase in depolarization on the right side of the impact point. This is only observed when the outer facing cells of an onion layer are used and not the core facing side. Multiple examples of this can be found in the supplementary materials to this manuscript. This suggests some dependence to the physiologic structure of the sample that will be studied in detail in future works. To relate the observed changes to the plasma interaction, time-resolved images of the Mueller matrix have to be obtained during the plasma impact.

### Approach 2: In-situ examination during plasma exposure

The second approach is followed to characterize the plasma-surface interaction that has induced the changes observed in Fig. 3, by obtaining the experienced electric field. This is done by applying Mueller polarimetry in-situ during plasma exposure and analyzing the two obtained time-resolved Mueller matrices.

The acquisition of the first Mueller matrix is done time-resolved to capture the optical characterization of the combined sample after an ionization wave has impacted the surface and thus temporarily deposited charges and electric fields are present. This is done by using a time delay relative to the trigger signal (i.e. the rise of the AC sine-wave) of 7 $\upmu$s. The total acquisition of the Mueller matrix takes approximately 130 s.

In previous works^31,32^, with only the electro-optic BSO material, it was shown that charges are temporarily deposited each AC period between the moment of impact (at 3–4 $\upmu$s relative to the rise of the applied voltage) until the polarity of the applied voltage changes and the negative half period starts at 16.7 $\upmu$s. Thus a time delay of 28 $\upmu$s is taken for the second Mueller matrix to obtain the optical properties without any influence of electric field.

**Figure 4:**
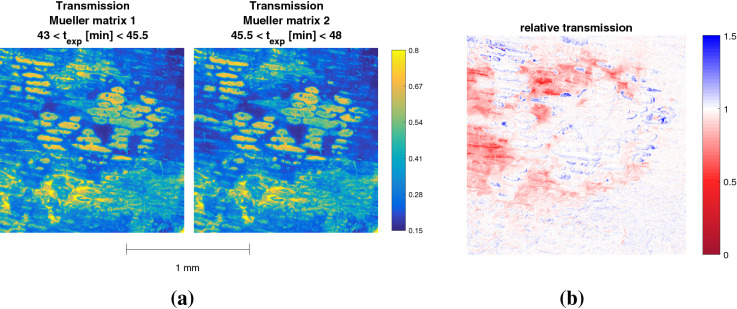
The transmissions of the first and second Mueller matrices, show in (**a**), measured time resolved under continious plasma exposure with the accumulated exposure time indicated by $t_{exp}$. The relative change of transmission between the two is shown in (**b**).

The transmission of the two Mueller matrices is shown in Fig. 4a. The two matrices were taken after 43 and 48 min of exposure to plasma respectively, denoted here by $t_{exp}$. The two transmission images do not seem to be distinctively different from each other. However, when the relative transmission is taken through division, some changes become evident shown in Fig. 4b. Areas where the relative transmission is smaller than one, i.e. shown in red, indicate that the transmission from the second Mueller matrix has decreased compared to the first one. This could be a result of a further and severe dewetting of the sample or could indicate a (chemical) surface change. In the center of the image the relative transmission appears to be zero, indicating no change has occurred.

**Figure 5:**
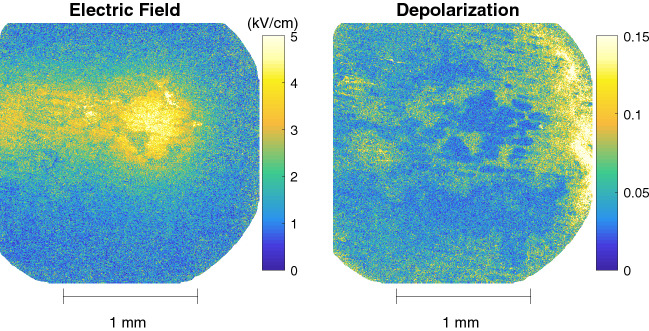
The electric field pattern (kV/cm) obtained by analyzing the change in linear retardance, together with the total depolarization using the $L_u$ matrix diagonal elements. The results portray the surface conditions of plasma exposure during the 43 and 48 min of treatment.

The plasma-surface interaction is characterized by obtaining the electric field through the linear birefringence. This is retrieved by applying the logarithmic decomposition to the two time-resolved Mueller matrices to obtain the associated $L_m$ and $L_u$ matrices which contains the disentangled optical properties and the depolarization information respectively. The electric field is derived from the observed changes in linear birefringence when charges are present (first time-resolved matrix) and when they are not (second time-resolved matrix). Birefringence is evaluated from elements (2,4) and (3,4) of the respective $L_m$ matrices. The total depolarization is obtained from the diagonal values of the $L_u$ matrix and they are corrected for the values detected when only BSO (without onion cells) is examined to focus on the increase of depolarization caused by the addition of the complex sample. The obtained electric field and depolarization is shown in Fig. 5. Both the Mueller matrices and the logarithmically decomposed $L_m$ and $L_u$ matrices can be found in the PhD thesis of Slikboer^25^.

Electric field values are observed with a maximum of 5 kV/cm. This is located in the impact point of the ionization waves to the surface. The plasma jet is oriented on the right-hand side at a 45 degree angle meaning the surface propagation that follows the impact is towards the left. The surface charges that are deposited during the surface propagation cause a lower electric field than the charges deposited in the impact region.

In the impact region the depolarization suggests that the onion cells have been completely removed since the total depolarization is close to zero. Around the impact point the depolarization has values of approximately 0.07. This means that apparently etching processes have not fully removed the onion cells there at these timescales. The same holds with the area left of the impact point where the surface propagation takes place. This overlaps with the electric field where the values are lower, approximately 3 kV/cm. This corresponds also with the area indicated in Fig. 4b to a decrease in relative transmission between the two Mueller matrices.

On the right edge of the depolarization image there is a distinct increase, similar as observed in Fig. 3. Since the electric field values are zero in this area we can conclude that these changes cannot have been caused by charged particles. Due to the orientation angle of the plasma jet, this area is likely to contain a much greater fraction of air than helium, meaning oxygen or nitrogen radicals could play an important role. A second diagnostic method is needed to examine what these changes of depolarization withhold, e.g through IR absorption spectroscopy.

**Figure 6:**
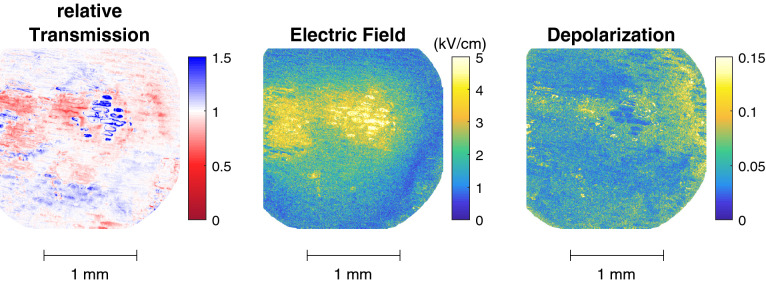
The relative transmission, induced electric field and total depolarization obtained from two time-resolved Mueller matrices measured between 11 and 16 min of accumulated plasma exposure.

Figure 6 shows the relative transmission, induced electric field and total depolarization obtained from two Mueller matrices that were measured during earlier plasma exposure of the same sample, i.e. between 11 and 16 min of accumulated plasma exposure. The results from other time intervals can be found in^25^. It can be seen that the plasma-surface interaction characterized by the electric field is of a similar nature. The surface evolution however is slightly different, indicated by the depolarization and relative transmission. Areas where the relative transmission is greater than 1, inside the impact region, indicate that etching processes are taking place within this exposure period. When etching has taken place, the total optical path has been reduced which means the transmission has increased.

## Discussion

Mueller polarimetry has been proposed as a novel optical diagnostic technique to study the plasma-surface interaction. It has been applied to simultaneously monitor the surface evolution of a complex sample while characterizing the plasma-surface interaction. The latter is done by imaging the electric field inside the electro-optic target on which the complex sample is placed. The examined complex sample in this work was a single layer of onion cells. This was chosen due to simplicity to prove the concept of the examination and separation of birefringence from depolarization. In the future this diagnostic allows for the investigation of more complex organic tissues, cells or membranes under plasma exposure.

The electric field pattern shown in Figs. 5 and 6 is induced by charge deposition during this surface interaction on top of the onion cell layer. It shows great resemblance with the patterns that have been examined in previous studies in single BSO crystals without the presence of the complex target^31,32^. Those results were obtained with a relatively more simple Sénarmont setup. That setup is specific to detect the retardance associated to the action of an electric field, however cannot be used when other properties such diattenuation and depolarization are present. In this work we present for the first time a general approach based on the Mueller matrix formalism which considers all possible polarimetric properties and which allows the study of complex samples.

The field values deduced from the linear birefringence in Figs. 5 and 6 correspond to the electric field strength inside the BSO crystal. This field is induced by the charge deposited on the surface since the influence of the electric field due to gas volume charges are limited as we have shown in previous work^10^. The field strength measured depends on the field penetration through the onion tissue and the BSO crystal. Therefore, the field values at a given position are not only depending on the local charge surface density but also on the thickness of the onion tissue (possibly modified by etching of the plasma) and its permittivity. Although the absolute value of the induced field in onion cells can therefore not be known, the pattern of the measured field nevertheless contains valuable information for understanding the plasma surface interaction. The pattern of the measured field gives an accurate image of the area where charged species have played a role on the surface reactivity. Additionally, the very short lifetime of charge species in the gas phase insure that the place where surface charge are detected correspond to the limit of gas phase ions production by the ionization wave. This is already a valuable indication for differentiating the chemistry induced solely by neutral species (radicals and stable molecules) from the processes involving ions reactivity and/or surface.

**Figure 7:**
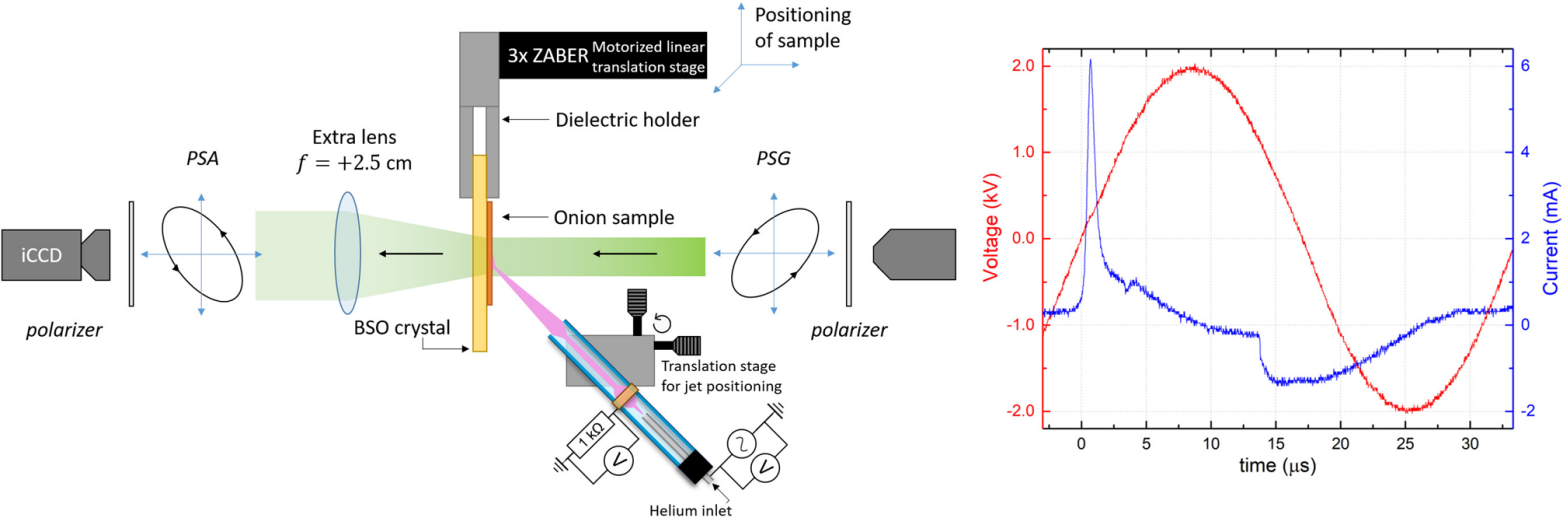
The Mueller polarimetry setup used to optically examine the electro-optic BSO material with a single layer of onion cells on its surface under exposure of a coaxial kHz-driven plasma jet (oriented at 45 degree at 7 mm distance). Polarized light coming from the *PSG* passes the sample towards the *PSA* before its captured by the iCCD detector. The voltage and current waveform obtained when the plasma jet is operational is shown on the right (30 kHz AC frequency with 2.0 kV amplitude). More details on the plasma jet operation is given in the text.

The implications of the latter can clearly be seen in Figs. 5 and 6, where an area with large depolarisation enhancement is observed on the right of the impact point where absolutely no electric field is measured. This shows a significant modification of the surface structure at a location where no charge species could have contributed. The helium flow used to feed the plasma jet is impacting at 45$^o$ from the right to the left on Fig. 7. Therefore, the strong depolarization signal observed occurs only in an area with low helium density in the gas, but in the vicinity of the impact point within a radius of less than 1 mm suggesting an impact of excited species produced from N$_2$ and O$_2$. Regions where charged species have been deposited show indications of etching. Inside the impact point where the detected field is relatively high (5 kV/cm) etching occurred fast and fully removed the onion cells. In the “tail” area of the surface propagation behind (towards the left) of the impact point the amount of deposited charges was less, indicated by a lower detected electric field (3 kV/cm), and hence the etching was less prominent.

Our diagnostic and approach presented in this work provides an important tool since in the last decades non-thermal atmospheric pressure plasmas have been applied in the field of plasma medicine and plasma agriculture without a clear method of characterizing information relating the intrinsic plasma-surface interaction. Often the observed material changes or biomedical treatment are reported with only the operation settings of the plasma (i.e. voltage, gas flow, distance, etc) while nothing is known of the interaction. By comparing the electric field induced in an electro-optic material used as examination slide, the conditions are examined to which a sample (with high dielectric constant similar as BSO) is exposed to. At present and despite the existence of active research in the field of plasma physics and related applications, there is still some lack of knowledge on either the plasma properties close of the target depending of the type of plasma and also on the details of the interaction of the plasma with the target itself. Our method represents an efficient and systematic way to characterize the plasma properties (electric field) and also the interaction of the plasma on a target (for instance, dewetting, etching, etc). Given the generality of the Mueller matrix approach, the method can be applied to any type of sample, provided that measurement could be done in transmission, which is required to be able to apply the logarithmic decomposition.

The electric field reported in this work is not necessarily the electric field experienced by the complex organic sample as well. This depends on the dielectric properties of the sample and its thickness. It has been shown by modeling that the electric field obtained from the BSO target is comparable to other targets in the absence of grounded planes with similar thickness (0.5 mm) and a permittivity higher than 4^17^. When cold plasma jets are used for e.g. biomedical treatment of temperature sensitive tissues or surface functionalization of polymers, many types of targets meet these conditions. When the dielectric constant is lower, the electric field is not only determined by the surface charges but also due to the volume charges of the plasma in the gas phase^10^. Nonetheless, as shown in this work, the electric field captured inside the BSO target can be used to locate the plasma interaction and compare the charge deposition in different areas, while monitoring the surface evolution of the sample.

The proposed method provides interesting quantitative information about the effects of plasma interaction with the sample, which can be combined with measurements done with other techniques, i.e. IR spectroscopy or electron microscopy to get much more richer details about chemical and micro-structural modifications produced by the plasma in the target.

## Methods

### Experimental setup

The experimental setup consists of the polarimeter used to investigate the plasma surface interactions occurring with complex targets and the atmospheric pressure plasma jet, both are shown in Fig. 7. The setup resembles earlier configurations that were used to study solely the electro-optic targets under plasma exposure^8–10^. A 2.5 cm collimated light beam originating from a white LED light source (Thorslabs MCWHL5-C1) with total beam power of 440 mW passes a 10 nm colorfilter at 530 nm. Afterwards it passes sequentially a linear polarizer, the *Polarization State Generator* (PSG), the sample, the *Polarization State Analyzer* (PSA) and a final linear polarizer before the intensity is imaged by the iCCD camera (Princeton Pi-Max4). The intensity which is captured at the iCCD camera depends on the optical responses of the elements within the PSG and PSA and on the optical properties of the sample. The sample in this case consists of an electro-optic BSO (Bi$_{12}$SiO$_{20}$) crystal on which on top a single layer of onion epidermal cells is placed. The sample is under exposure of an atmospheric pressure plasma jet which is introduced later.

The PSG and PSA consist each of two ferro-electric liquid crystals with a respective retardance of a quarter wave and a half wave at the working wavelength. The orientation of each liquid crystal can be switched automatically between a negative and a positive state using a small external driving DC voltage. As a result, 4 different polarization states can be generated and analyzed in order to obtain 16 different intensity images at the iCCD camera. These intensity images form the $4\times 4$ intensity matrix that is used to construct the $4\times 4$ Mueller matrix of the examined sample. This is possible because the Mueller matrices of the PSG and PSA are known and obtained through the eigenvalue calibration procedure^33,34^. A more detailed explanation of the experimental setup can be found in^25^.

A +2.5 cm lens is placed in front of the sample to study the complex micro-structure of the onion cells with high resolution and to increase the numerical aperture of the polarimeter which allows the detection of scattering of light through depolarization. Scattered and direct light are detected simultaneously and incoherently by the camera. Since the polarization state of direct and scattered light is in general different they contribute to generate not fully polarized Mueller matrices.

With our Mueller polarimeter the Mueller matrix and thus the optical properties can be examined time-resolved. This is possible because the switching of the optical states and the acquisition at the iCCD camera is controlled with respect to an external trigger signal. This signal relates to the applied voltage cycle at the plasma jet and hence to the generation of the ionization waves and the induced plasma-target interaction. Since a reproducible plasma interaction is examined, the intensity images are obtained during multiple voltage cycles. The time delay of the acquisition can be varied to do time-resolved optical measurements of the Mueller matrix that correspond to the optical properties of the examined material before, after and/or during the impact of the ionization waves at a sub-$\mu$s timescale following the kHz-driven frequency range of the applied voltage cycle. To the best of our knowledge, this makes it the only Mueller polarimeter with the capability to do time-revolved measurements of samples with (reproducible) time-dependent optical characteristics.

### Analysis of Mueller matrices

The measured Mueller matrices are normalized by dividing the matrices by their first top left element. This element represents the overall transmission of the examined sample to unpolarized light. The normalized matrix contains all the polarimetric characteristics regarding diattenuation, birefringence, and depolarization. These characteristics are further divided for circularly polarized light and linearly polarized light along the 0/90 degree axes and the 45/135 degree axes. The polarimetric characteristics are entangled within the Mueller matrix and to analyze them a decomposition is required^35^. Since the measurements in this work are performed in transmission, the logarithmic decomposition is used because the measured polarimetric properties of the sample are the result of the cumulative optical response of the sample^36–38^.

When depolarization is present, the average polarization properties (diattenuation and birefringence) can be separated from the respective depolarization ones. To do so the logarithm of the Mueller matrix is separated in two matrices, called $L_m$ and $L_u$^39^. From the birefringence the electric field is retrieved similar as done in previous works^8^. The total depolarization is retrieved from the three depolarization elements within the $L_u$ matrix as is conventional.

### Atmospheric pressure plasma jet

The atmospheric pressure plasma jet used in this work generates *cold* ionization waves by applying a AC sine wave with a frequency of 30 kHz and amplitude of 2.0 kV. The plasma is considered cold/non-equilibrium since the heavy particles remain at low temperatures while the electrons go to elevated energies. The voltage is applied to a stainless steel tube which is placed within a dielectric capillary (inner diameter 2.5 mm and outer 4.0 mm), see Fig. 7. A grounded copper ring is attached 5 mm downstream from the end of the powered electrode, leaving 20 mm from the grounded electrode to the end of the capillary. Helium flows at 1 slm from the powered electrode through the capillary into an open environment where it mixes with air at atmospheric pressure. This plasma jet in coaxial configuration generates one ionization wave each voltage cycle during the positive half period^31,40^. The ionization wave propagates from the powered electrode underneath the (shielded) grounded ring towards the end of the capillary from where it propagates in the *plasma plume* area before interaction with a target which is placed at a 7 mm distance. The electrical characterization of this plasma jet is reported in^40^ and shows a power dissipation of 0.5 Watt. The plasma jet is oriented at a 45 degree impact angle towards the targeted sample (at 7 mm distance) to not block the polarized light beam of the polarimeter, as shown in Fig. 7. Depending on the impact angle, a surface discharge on the dielectric target can initiate behind the impact point at the surface in the direction of the helium flow^41^. The current profile shows a maximum of 6 mA at the moment when the ionization wave is still inside the capillary tube, accumulating charge beneath the grounded ring. Interaction between the ionization wave and the targeted surface is indicated by the small current “dip” at approximately 4 $\mu s$. This indicates the time when charge deposition starts to occur. Charges remain stable on the surface until the current profile changes at approximately 14 $\mu s$^31^.

### Experimental approaches

The extended examination of the combined sample consisting of the electro-optic BSO (Bi$_{12}$SiO$_{20}$) target with the complex sample is done using the polarimeter following two approaches, shown in Fig. 1b. The first (shown in green) corresponds to the optical measurement of the Mueller matrix before and after a plasma exposure. This allows to investigate the changes that the targeted material has undergone. Since no plasma exposure is applied during these measurements, the optical response of the electro-optic crystals is minimal and the optical properties that are detected relate to the complex sample. A mapping of the examined target can be done by scanning the targeted material along a predefined grid using the ZABER motorized translations stages. This allows to increase the field-of-view and see the extend of the surface interactions.

The second approach (shown in Fig. 1b in red) relates to the in-situ investigation of the interaction using time-resolved measurements of the Mueller matrix of the combined sampled during continuous operation of the plasma jet. The AC sine wave voltage cycle is used as a trigger signal to perform time resolved measurements that relate to the periodical optical state before and after an ionization wave has impacted.

The acquisition of a time-resolved measurement takes approximately 130 s, since multiple exposure of 3 $\upmu$s are taken to reduce the influence of noise. Since two time-resolved measurements are needed (rel. before and after impact) and an initiation time of the plasma jet is necessary, every plasma exposure is applied continuously for 8 min. The sample is repetitively exposed and measured following both approaches for several series, up to a total exposure time of 48 min, after which a new sample is placed and examined for reproducibility.

The optical properties measured according to the second protocol relate to both the electro-optic properties of BSO and of the complex sample. Only repetitive changes between the time-resolved measurements are expected within the birefringence that relate to the retardance induced by electric fields generated due to temporarily deposited surface charges.

The evolution of the targeted sample can cause errors for the optical properties (from which the electric field is determined) when they occur on timescales similar or smaller than the total acquisition of the intensity matrix. The measurements performed following approach 1 can be used to verify whether this is the case.

It was observed with initial testing that when a “*fresh*” and newly prepared sample of a single onion cell layer was used, that during the first minutes of plasma exposure significant changes were observed. This rapid evolution of the sample structure and the related optical response rendered it impossible to measure reliably the Mueller matrices of the combined sample during that time because the sample properties evolve drastically while the different images needed to obtain a Mueller matrix are acquired. Therefore it was decided that during the first minutes of plasma exposure to a new sample, simply the intensity of light reaching the iCCD camera was monitored in time, with the liquid crystals in the PSG and the PSA left in a neutral orientation (shown in Fig. 1b in blue). Although no quantitative polarimetric information is not obtained, it allows visualization of the changes that occur during these first minutes.

## Supplementary material

Supplementary information
