# Supplementary material for: In-situ monitoring of an organic sample with electric field determination during cold plasma jet exposure
Source: Sci Rep. 2020 Aug 12;10:13580. doi: 10.1038/s41598-020-70452-w (PMC7423608; doi:10.1038/s41598-020-70452-w)
Supplement: Supplementary file 1 — Supplementary information [file 41598_2020_70452_MOESM1_ESM.pdf]

Supplementary Figures to: In-situ Monitoring of an Organic Sample with Electric Field Determination during Cold Plasma Jet Exposure

Elmar Slikboer[1,2,3], Ana Sobota[2], Enric Garcia-Caurel[3], and Olivier Guaitella[1*]

[1]LPP, CNRS, Ecole Polytechnique, Sorbonne Universite, IP–Paris, 91128 Palaiseau, France
[2]Department of Applied Physics, EPG, Eindhoven University of Technology, The Netherlands
[3]LPICM, CNRS, Ecole Polytechnique, IP–Paris, 91128 Palaiseau, France
[*]olivier.guaitella@lpp.polytechnique.fr

ABSTRACT

Figures 1 and 2 show the mapping of the optical properties of various samples that have been exposed to a plasma jet for a significant amount. They are supplementary to figure 3 of the main manuscript. As discussed in the main manuscript, each mapping consist of nine (sub)images. The center image locates the main interaction area of the plasma jet with the single layer of onion cells. The same acquisition settings and data treatment is used as discussed in the main manuscript. The maps shown in figure 1 and 2 are obtained after minimally 40 minutes of plasma exposure. The transmission and linear retardance show the etching and drying of the sample around the impact area. A significant increase of depolarization is observed on the right side of the impact area, when cells are taken from the outer face side of an onion slice. When a single layer of onion cells from the core facing side is exposed to plasma this increase of depolarization is not observed. As explained in the manuscript, these mapping results are obtained after plasma exposure. The results obtained *during* plasma exposure (i.e. showing the induced electric field) for the additional samples shown in these supplementary figures can be found in the appendix of the PhD Thesis of Slikboer[1].

References

1. Slikboer, E. T. *Investigation of Plasma Surface Interactions using Mueller Polarimetry*. Ph.D. thesis, Eindhoven University of Technology, École Polytechnique, Université Paris-Saclay, ISBN 978-90-386-4634-3, research.tue.nl/en/publications/investigation-of-plasma-surface-interactions-using-mueller-polari (2018).

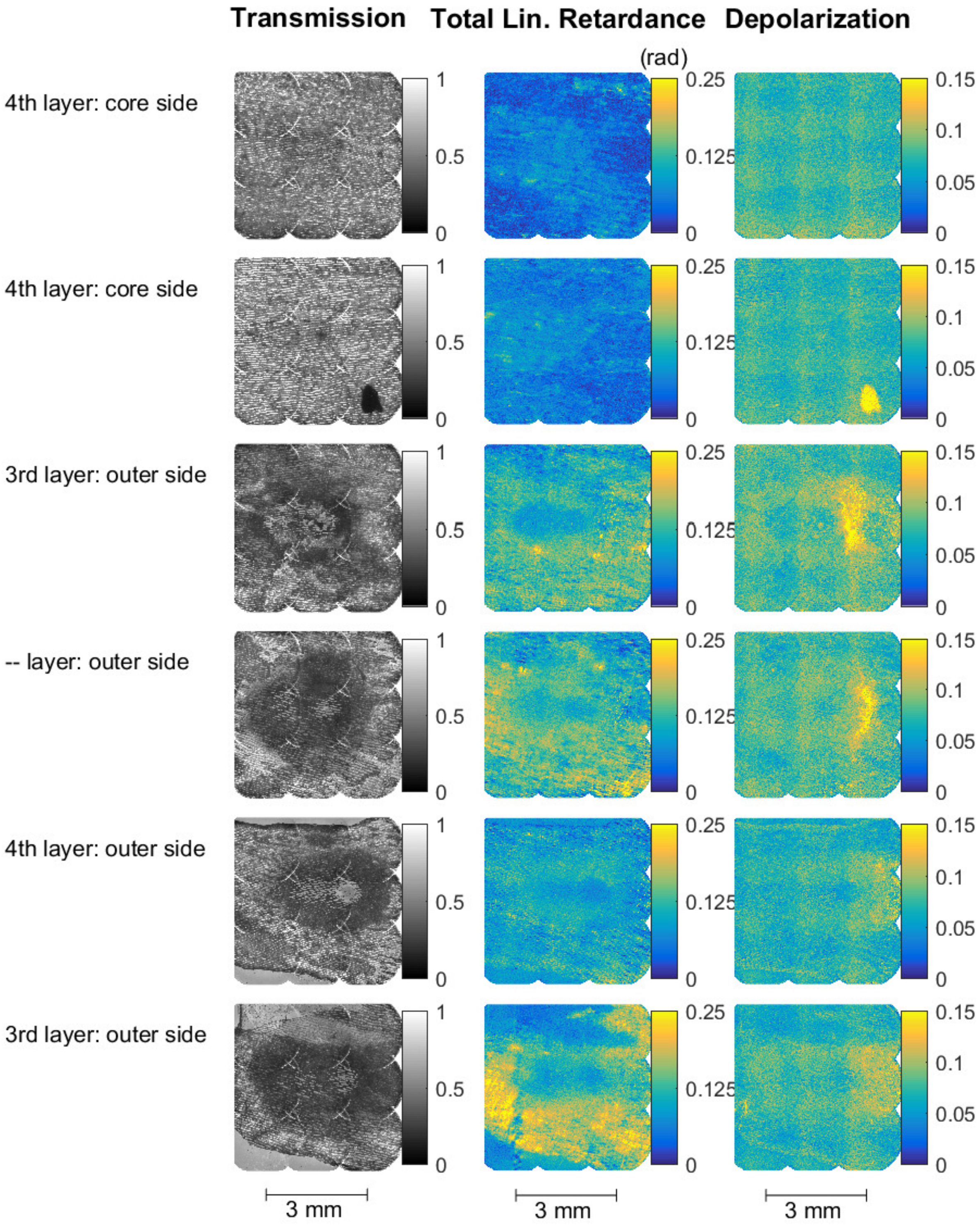

Figure 1. This supplementary figure shows the optical properties (transmission, total linear retardance, and depolarization) of various samples of a single layer of onion cells. The cells were taken either from the core facing side or the outer facing side of an onion layer.

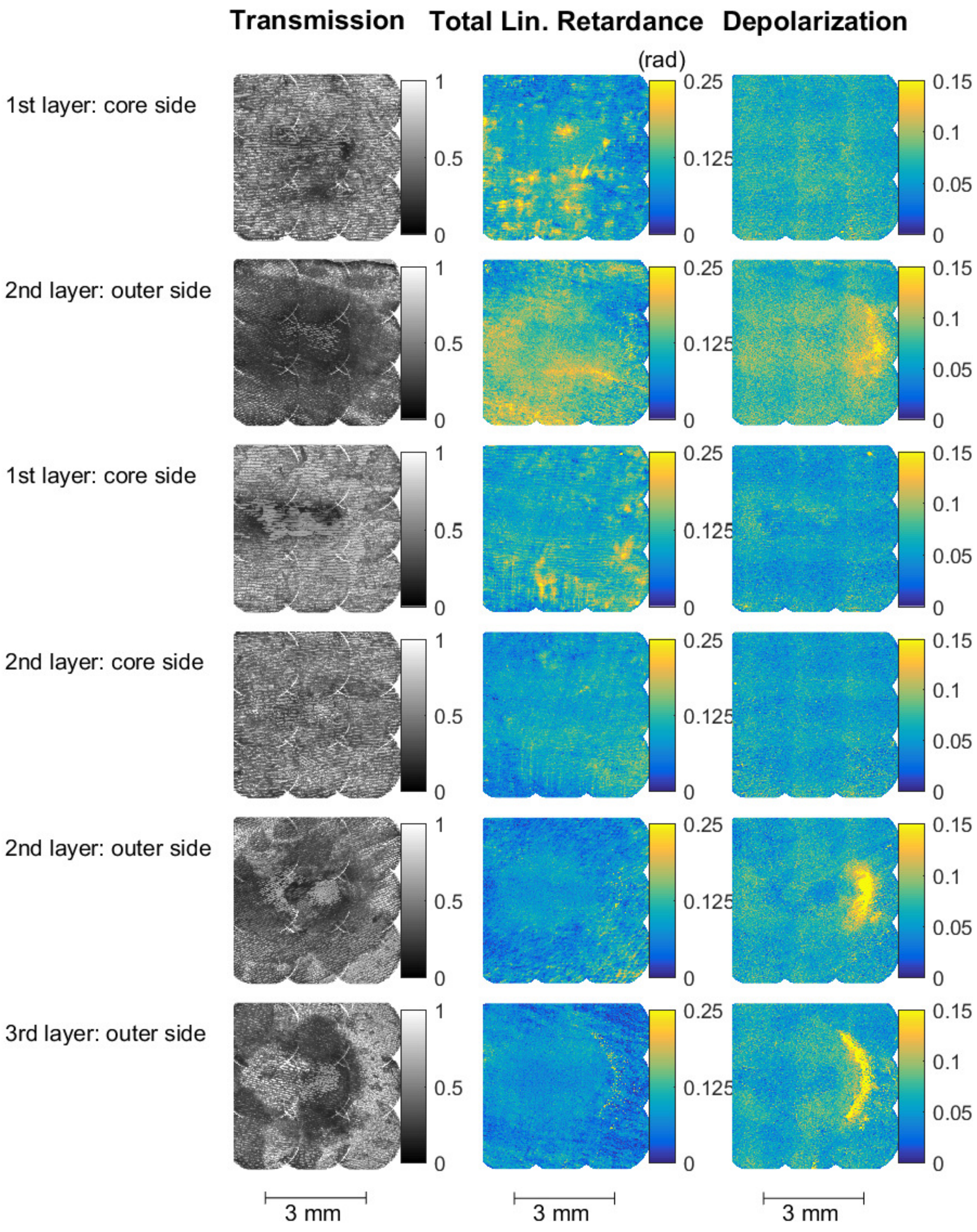

Figure 2. This supplementary figure shows the optical properties of additional samples to figure 1.
